# Factors influencing the use of antenatal care in rural West Sumatra, Indonesia

**DOI:** 10.1186/1471-2393-12-9

**Published:** 2012-02-21

**Authors:** Yenita Agus, Shigeko Horiuchi

**Affiliations:** 1 St. Luke's College of Nursing, Maternal Infant Nursing and Midwifery, 10-1 Akashi-cho, Chuo-ku, Tokyo 104-0044, Japan; 2 Syarif Hidayatullah State Islamic University, Faculty of Medicine and Health Science, Jl. Kertamukti Pisangan Ciputat, Jakarta, Indonesia

**Keywords:** Pregnant women, Traditional birth attendant and traditional beliefs

## Abstract

**Background:**

Every year, nearly half a million women and girls needlessly die as a result of complications during pregnancy, childbirth or the 6 weeks following delivery. Almost all (99%) of these deaths occur in developing countries. The study aim was to describe the factors related to low visits for antenatal care (ANC) services among pregnant women in Indonesia.

**Method:**

A total of 145 of 200 married women of reproductive age who were pregnant or had experienced birth responded to the questionnaire about their ANC visits. We developed a questionnaire containing 35 items and four sections. Section one and two included the women's socio demographics, section three about basic knowledge of pregnancy and section four contained two subsections about preferences about midwives and preferences about Traditional Birth Attendant (TBA) and the second subsections were traditional beliefs. Data were collected using a convenience sampling strategy during July and August 2010, from 10 villages in the *Tanjung Emas*. Multiple regression analysis was used for preference for types of providers.

**Results:**

Three-quarter of respondents (77.9%) received ANC more than four times. The other 22.1% received ANC less than four times. 59.4% received ANC visits during pregnancy, which was statistically significant compared to multiparous (*p *= 0.001). Women who were encouraged by their family to receive ANC had statistically significant higher traditional belief scores compared to those who encouraged themselves (*p *= 0.003). Preference for TBAs was most strongly affected by traditional beliefs (*p *< 0.001). On the contrary, preference for midwives was negatively correlated with traditional beliefs (*p *< 0.001).

**Conclusions:**

Parity was the factor influencing women's receiving less than the recommended four ANC visits during pregnancy. Women who were encouraged by their family to get ANC services had higher traditional beliefs score than women who encouraged themselves. Moreover, traditional beliefs followed by lower income families had the greater influence over preferring TBAs, with the opposite trend for preferring midwives. Increased attention needs to be given to the women; it also very important for exploring women's perceptions about health services that they received.

## Background

According to the United Nations Millennium Development Goals [1], every year, at least half a million women and girls needlessly die as a result of complications during pregnancy, childbirth or the 6 weeks following delivery. Almost all (99%) of these deaths occur in developing countries.

Reproductive health is a priority program for all the Southeast Asia Regional countries, including Indonesia, a developing country, where maternal mortality continues to be a major problem. According to a World Health Organization (WHO) report [2], the Indonesia Maternal Mortality Rate (MMR) continues in the high range of 420/100,000 live births, while coverage of births assisted by skilled providers is still low. The deaths are due mainly to five major causes: hemorrhage; followed by eclampsia, infection (sepsis), abortion complications and obstructed labor [3]. Other issues compounding maternal mortality are lack of knowledge and preparedness about reproductive health in the family, community and health providers. Also there are inadequate numbers of reproductive health specialists to manage complicated pregnancies and deliveries and there is inadequate quality and access to all levels of obstetric care and other reproductive health service.

To prevent unwanted outcomes of pregnancy, antenatal care (ANC) is the most important method for detecting pregnancy problems in the early period [4]. ANC is a critical element for reducing maternal mortality, and for providing pregnant women with a broad range of health promotion and preventive health services [5]. One of the most important functions of ANC is to offer health information and services that can significantly improve the health of women and their infants. ANC is also an opportunity to inform women about the danger signs and symptoms for which immediate assistance should be sought from a health care provider [6]. The numbers of ANC visits vary depending on the country. In Western Europe, North America and many other countries ANC includes 12-16 visits to health care services by the pregnant woman, as well as provider visits to her home [7]. Even so, the WHO and the Indonesia government recommended a minimum of four ANC visits for a woman with a normal pregnancy; that is one visit each in the first and second trimester, and two visits in the third trimester (1-1-2 frequency). Unfortunately, the actual number of visits does not always reach the recommended number of minimum visits, signaling a need for closer examination of causal factors.

The number of ANC visits varies within Indonesia. Surveys indicated that the 1-1-2 frequency was more likely to be completed by urban women (72%) compared to rural women (57%) [8]. Based on WHO statistics [2], only 81% of the women in Indonesia attended the recommended four antenatal visits.

The percentage of pregnant women who received ANC services in Indonesia particularly on the island of Java (1997), receiving at least one visit, was higher (82.3%) compare to islands outside of Java (West Nusa Tenggara, Maluku and Irian Jaya) (65.9%). Half of the women received less than the 1-1-2 frequencies and of those only 60% received the standard care such as measurements of weight, height, blood pressure, weight of womb, tetanus toxin injection and given iron tablets [9].

A review of studies from various countries indicates that the ANC utilization rate is still low due to many factors that need to be examined such as socio-demographic features, knowledge of social support and ANC services [10]. Maternal and child welfare is not only related to health services provided by government and private organizations, it is also related to women as mothers including their education, economic status, culture, environment, and professional development [11]. For example, a study in Xien Khouang Province, Lao PDR [12] showed that significant predictors of ANC utilization were level of education, income, knowledge, attitude, distance to service, availability of public transportation, cost of transportation, and cost of services.

Furthermore, in many developing countries a Traditional Births Attendant (TBA) is still the dominant person, especially in rural areas. Tugiminize in Uganda [13] found that the TBA was popular with pregnant women for a number of reasons such as not being expensive, similar beliefs, tolerant and kind. In addition, some of the reasons for high antenatal visit drop-outs in Maluku, Indonesia were: women perceived the need for the midwife only if there was a problem; women were more comfortable with a TBA because she spent more quality time with them and used a barter system for payment; women feared they could not afford to pay the price of the health center midwives; and women didn't have confidence in the new village midwives because they were often young, unmarried, and inexperienced [14].

Inadequate access and under-utilization of modern health care services were major reasons for poor health in developing countries [15]. To improve planning and provision of ANC in a specific setting it is important first to be able to characterize those women and their families not receiving adequate care. Few studies with that perspective have been conducted in developing countries including Indonesia.

### Statement of the problem

ANC is a necessary component of maternal health in order to identify complications and danger signs during pregnancy. Regular ANC visits can provide some benefits for the women such as a strong relationship between women and the health care provider that can result in reducing complications during pregnancy. Women in rural areas in Indonesia tend to receive less ANC visits than urban women. This study was needed to find the key factors involved in why women in rural areas could not or do not go to ANC.

### Purpose

To describe the factors related to low utilization of ANC services among pregnant women in a rural area in Indonesia.

## Methods

### Design of the study

A descriptive design was used for identifying factors of why women do not take advantage of appropriate ANC services.

### Setting and samples

#### Setting

Tanah Datar is a regency (local government unit) of West Sumatera province of Indonesia. The regency is divided into 14 districts and has a total population of 334,000. Each district has a Public Health Centre (PHC) responsible for the sub districts and their village level midwifery clinic. The researcher selected the PHC *Tanjung Emas *based on convenience sampling. The research data was collected in 10 villages of the 14 villages of *Tanjung Emas*, during July and August 2010. The researcher invited 14 village midwives in this area and requested their cooperation. Four village midwives refused to participate in this study. Midwives distributed questionnaires after receiving training from the researcher.

#### Sample

Potential participants were 200 women selected under the following conditions: married women who were pregnant or had experienced birth; living in the village; able to communicate in the Indonesian language; and living with their nuclear family or extended family.

### Instrument

The researcher developed a questionnaire containing 35 items, based on the PRECEDE-PROCEED Model (PPM) [16]. It was translated from English into Indonesian as a language familiar to most Indonesians.

This questionnaire contains four sections. Section one and two included women's socio demographics factors such as age, living situation, health insurance status, education, mode of transportation to health care, distance to health care, employment and history of pregnancy.

Section three contained about women's basic knowledge of pregnancy questions. It consisted of eight items (see Table 1) scored as '1' for *yes *and '0' for *no*, yielding a maximum of 8 points to a minimum of zero. Each subject's total score was converted into a mean. The highest mean indicated superior knowledge, while the lowest mean was treated as inferior knowledge. For purposes of this study knowledge meant the fact or condition of knowing something with familiarity gained through experience.

**Table 1:** Basic knowledge of pregnancy questionnaire by number of women responding correctly in rural area of Indonesia, 2010 (N = 145)

Basic knowledge of pregnancy	No. of subject with the correct answer	%
Antenatal care is important to check my condition during pregnancy (true)	142	97.9
The first antenatal care examination must be done within the 3 months (true)	121	83.4
Eating more iron containing food during pregnancy can prevent anemia (true)	137	94.4
Pregnant women need a supply of calcium (true)	144	99.3
Pregnant women must have their blood pressure checked during pregnancy (true)	143	98.6
Headache is normal sign during pregnancy (false)	55	62
Pregnant women needs to go to hospital when they have high blood pressure (true)	130	89.6
Bleeding during pregnancy is common and you don't have to worry about it (false)	17	88.3

Section four contains two subsections. The first is about preferences for ANC (total of 22 items). Preference for TBAs consisted of 12 items and preference for midwives consisted of 10 items. Women were asked to indicate their preference using a 5-point Likert-type scale: 1) *strongly disagree*, to 5) *strongly agree*. The higher the points the more they preferred the TBA. Lower points indicated a preference for midwives. Several reverse items were included. To prefer a TBA meant that a woman was more likely go to a TBA for ANC rather than to a trained midwife. The second subsection asked about traditional beliefs related to ANC and consisted of five questions using a 5-point Likert -type scale. The higher score meant that women welcomed traditional beliefs from her community.

### Procedure

The researcher and trained assistant researcher distributed the questionnaires to the midwives in the villages who had recruited women meeting the eligibility criteria. The midwives provided them with the informed consent information.

Questionnaires and informed consent forms were collected by the midwives and returned to the assistant researcher.

### Data analysis

The questionnaire used in this study had 35 items developed from the PPM and an appropriate sample size of over 140. Non-parametric statistics: means, medians, quartiles and percentages were used for descriptions. Chi square, *T*-test and F-test were used for comparing knowledge scores, traditional belief scores, and TBAs scores and midwives or nurses scores between subgroups from different demographics and socio economic statuses. Multiple regression analysis was conducted with section four (preference for types of care provider). All tests were two tailed with a *p *value of < 0.05 considered significant. SPSS ver.17.0 was used for data analysis.

### Ethical consideration

The Ethics Review Board of St. Luke's College of Nursing Tokyo, Japan (approval number: 10-023) approved the study. In addition *Badan Kesatuan Bangsa, Politik dan Perlindungan Masyarakat *West Sumatera Province (N0.B.070/864/WAS-BKPL/2010) and *Badan Kesatuan Bangsa, Politik dan Perlindungan Masyarakat *Regency of Tanah Datar (No. 070/354/KBPLM/2010) provided approval.

## Results

Of the 200 questionnaires distributed, 147 (73.5%) were returned and after questionnaires with incomplete data were excluded, 145 remained for the final analysis. In keeping with the trend of previous studies about what influences antenatal clinic visits, items in the questionnaire are referred to as factors and should not be confused with the term factors resulting from scale development.

### Characteristics of women

The average age of the women was 29.7 years (*SD *= 5.6). The majority of the sample was age 21 to 34 (75.9%) and 22.1% of women 35 or older. All of the women were Muslim. Most of the women lived with their husbands (94.5%). Sixty-six percent of the women had more than two children and 31% were experiencing their first pregnancy. Living in an extended family were 52.4% and 47.6% lived in a nuclear family. A majority of the women (65.5%) finished secondary education and only 34.5% finished basic education. However, the majority of the women (74.5%8) did not work, and only 11% worked in the farming sector.

More than half of the women (55.9%) used public transportation for ANC visits. The average distance from their home to hospital was 1,570 m (*SD *= 2,009, *M *= 1,000). The number of women who were encouraged by their family to receive ANC (44.8%, *n *= 65) was almost same compared to those who encouraged themselves (55.2%, *n *= 80). Only 13.1% of respondent used ASKESKIN (health insurance for poor families) as their usual health care payment and the majority were self pay (86.9%). Midwives assisted 65.5% of the women while TBAs assisted only 3.4%. The government hospital was chosen for delivery by (44.1%), followed by village midwives practice (29.0%), and private hospitals (18.6%). There were no statistically significant differences comparing women's socio-demographic.

### Scores of knowledge, traditional belief, preference for TBAs and preference for midwives

The questionnaire used in this research had four sections; (1) knowledge, (2) traditional beliefs, (3) preference for TBAs and (4) preference for midwives. The average mean score of basic knowledge was 7.1 out of 8 (*SD *= 8.8) range of 5-8. Traditional belief mean score was 10.31 out of 25 (*SD *= 2.61) range 5-21. Preference for TBAs mean score was 25.52 out 60 (*SD *= 7.25) with a range 12 to 50. Preference for midwives mean score was 36.84 out 50 (*SD *= 4.45) with a range 14 to 46.

### Basic knowledge of pregnancy

The majority of subjects scored highly in the knowledge section (Table 1). Most of the subjects gave the correct answer to items on basic health knowledge. In seven out of eight items, women answering correctly was over 80%: "ANC is important", "eating iron containing food prevents anemia, "pregnant women need calcium", "pregnant women must have their blood pressure checked", "pregnant women need to go to the hospital if they have high blood pressure" and "bleeding is a normal sign during pregnancy (false)". However, for the item "headache is normal sign during pregnancy (false)", only 62% answered correctly.

### Distribution of ANC visit during pregnancy

The Indonesia government recommends ANC four times during pregnancy. Nearly 80% (77.9%) of respondents received ANC more than four times. The other 22.1% received ANC less than four times.

The researcher divided the sample into two groups for analysis: women who had less than four ANC visits and women who had four or more ANC visits. Women aged 21 to 34 were more likely to receive less than four ANC visit (87.5%) compared to those who were over 35 years old. As a group primiparous received less than four ANC visits, which was statistically significant compared to multiparous (*p *= 0.001). Women who finished basic education tended to receive fewer than four ANC visits compared to women who finished secondary education (*p *= 0.07). Women who accessed ANC services by walking were more likely receive less than four ANC visits compared to women who used transportation (*p *= 0.069). See Table 2 for specific details.

**Table 2:** Distribution of ANC visits during pregnancy by socio-demographic characteristics in rural area of Indonesia, 2010 (N = 145)

		Less than 4 visits	4 or more visits	
			
	Total N = 145 (%)	*n = 32*	*n = 113*	*p *value
Characteristic				
Age (years) %				
< 20	3 (2.1)	0	3 (2.7)	
21-34	110 (75.9)	28 (87.5)	82 (72.6)	0.193
> 35	32 (22.1)	4 (12.5)	28 (24.8)	
Parity				
Primiparous	49 (33.8)	19 (59.4)	30 (26.5)	0.001
Multiparous	96 (66.2)	13 (40.6)	83 (73.5)	
Mother's education				
Basic education	50 (34.5)	15 (46.9)	35 (31.0)	0.070
Secondary education	95 (65.5)	17 (53.1)	78 (69.0)	
Encouraged to get ANC				
My self	80 (55.2)	15 (46.9)	65 (57.5)	0.318
My family	65 (44.8)	17 (53.1)	48 (42.5)	
Usual source of payment				
Self pay	126 (86.9)	29 (90.6)	97 (85.8)	0.569
ASKESKIN	19 (13.1)	3 (9.4)	16 (14.2)	
Transportation mode to ANC				
I walk	64 (44.1)	19 (59.4)	45 (39.8)	0.069
Public transportation	81 (55.9)	13 (40.6)	68 (60.2)	
Complication during pregnancy				
Yes	34 (23.4)	8 (25)	26 (23)	0.816
No	111 (23.4)	24 (75)	87 (77)	

No relationship was found between parity and demographic factors such as educational background, economic status, age and distance from hospital.

### Factors related to the family income

The average monthly family income was Rupiah (Rp.) 831,034 (*SD *= 587,316) (1 USD = Rp. 9,070). This was lower than the minimum wage of West Sumatra (Rp. 1,055,000).

Among the three factors which were significantly different we found that: 1) The basic education group had lower incomes than the secondary education group (*M *= Rp. 591,000, *SD *= 343,99; *M *= Rp.957,368, *SD *= 679,954, respectively, *t *= 3.5, *p *= < 0.001), 2) Women who walked to receive ANC had lower income compared to the other group (*M *= Rp.703,125, *SD *= 486,718, *M *= Rp.932,098, *SD *= 678,753, respectively, *t *= 2.27 *p *= 0.024),3) Not surprisingly, women who used ASKESKIN as their usual source of payment revealed significantly lower incomes compare to women who were self pay (*M *= Rp.407,894, *SD *= 235,267; *M *= Rp.894,841, *SD *= 624,462, respectively, *t *= 3.35, *p *= 0.001).

### Factors related to preference for TBA

Table 3 shows the scores of preference for TBA which were analyzed between women's choice about delivery settings. It was found that women going to TBA ("yes" group) (*n *= 32) had a significantly higher mean score than the "never" group (*M *= 31.34, SD = 7.49; *M *= 23.87, SD = 6.30, respectively, *t *= -5.66, *p *< 0.000). Women who chose the government hospital to deliver had a significantly higher scores than women who delivered at the village midwives practice and private hospital (*F *= 7.80, *p *= 0.001). In addition, women who delivered with the assistance of TBA, had a statistically significant higher score compared to the others *(t *= 4.61, *p *= 0.011).

**Table 3:** Factors related to preference for TBA in rural area of Indonesia, 2010 (N = 145)

Factors	n	Mean	SD	*t *value or *F *value	*p *value
Going to TBA for care					
Never	113	23.87	6.30	-5.66	0.000
Yes	32	31.34	7.49		
Type of Hospital					
Private hospital	27	22.19	8.14	7.80	0.001
Village midwives practice	42	23.86	5.08		
Government hospital	76	27.62	7.34		
Delivery assistance					
Doctor	37	24.35	7.15	4.61	0.011
Midwife	103	25.5	7.04		
TBA	5	34.6	7.26		

### Encouragement to receive antenatal care

*What factors were associated with encouraging women to receive antenatal care*? Data were divided into two groups based on who encouraged the woman to seek ANC services: self or family. The scores on the factors, knowledge, traditional belief, preference for TBAs and preference for midwives, were compared between women who encouraged themselves and those who were encouraged by their family. Knowledge and preference for TBA was not significant. Interestingly, women who were encouraged by their family to receive ANC had statistically significant higher traditional belief scores compared to those who encouraged themselves (*M *= 9.13, *SD *= 2.40), (*t *= 3.03, *p *= 0.003). On other hand, women who encouraged themselves to go to ANC had statistically significant higher score in preference for midwives compared to those who were encouraged by their family (*M *= 37.62 SD = 3.96; *M *= 35.89, *SD *= 4.85, *t *= 2.36, *p *= 0.019).

### Multiple regression analysis

The results of multiple regression analysis are presented in Table 4. Variables such as traditional belief, distance to hospital and family income were significantly related to a preference for midwives. Traditional belief made the greatest contribution (ß = -0.492, *t *= 7.01, *p *< 0.001). Women's age and knowledge were excluded.

**Table 4:** Multiple regression analysis of preferences for midwives and preferences for TBAs in rural area of Indonesia, 2010 (N = 145)

	Variable	**R**^**2**^	Beta	*t *value	*p *value
	Traditional belief		-0.492	-7.01	0.000
Preference for midwives	Distance to hospital	0.328	-0.176	-2.51	0.013
	Family income		0.149	2.14	0.034
	Traditional belief		0.432	5.93	0.000
Preference for TBAs	Women's age	0.263	-0.202	-2.77	0.006
	Family income		-0.169	-2.31	0.022

Three items associated with preference for TBAs were traditional belief, women's age and family income with traditional belief making the greatest contribution (ß = 0.432, *t *= 5.93, *p *< 0.001). This trend was the same as preference for midwives. Regarding the preference for TBAs, two items knowledge and distance to hospital were excluded.

The results showed that traditional belief and family income were influential for women who chose both preference for TBAs and preference for midwives.

## Discussion

### Antenatal care visit

The results showed that 77.9% women in the *Tanjung Emas *area received more than the recommended minimum number of pregnancy ANC visits, which was close to the national average of 81% [2]. Compared to the Indonesia Demographics Health Survey [17], 75.5% of women in rural areas received ANC more than four times, whereas in urban areas it was 89.9%. Our study showed the same trend as the national survey. Other studies found a somewhat different trend. While, Doctor [18] reported that in Nigeria urban women also attended more ANC visits (82%), than rural women (45%) Pallikadavath [19] found that in India about 60% of rural women did not receive any ANC checkups during their last pregnancy.

Our results indicated that the main predictor of receiving less than four ANC visits was parity. However, these results are contrary to previous studies. Women with two or more children receiving antenatal care were 20-40%; lower compared to women with one child; [19] likewise Trinch [20] found that women with three or more children utilized less ANC. Similarly, Doctor [18] also reported that women who had given birth to one child had more ANC attendance than women with six or more children. However, high parity was also found to be significantly higher in women who did not attend ANC [21].

One possible explanation for the low ANC attendance among primiparous was a lack of understanding concerning the importance of ANC during pregnancy. This study examined attendance only by primiparous or multiparous rather than by specific number of children.

An interesting finding was mothers with a low education level were more likely to receive less than four ANC visits, although it was not statistically significant after adjusting for other factors. One reason was a small sample size. Taguchi's [22] study pointed out that higher education levels of women were associated with greater use of antenatal care in Indonesia Similarly, women's education consistently showed a positive association with antenatal care check-ups through visits to a health facility in all the states in India [19].

### Basic knowledge of pregnancy

Many respondents misunderstood the falsely worded item that "headache is normal sign during pregnancy". Likewise, in China [23] most women had knowledge about maternal health care but some items they missed. For the item "should pregnant women often check blood pressure", the majority answered correctly. Koblinsky [24] reported that in Bolivia, Indonesia and Pakistan the knowledge of danger signs was generally low; only 2-7% of women knew that eclampsia (convulsions) was a complication. In Gambia only 24.6% pregnant women were aware that hypertension and, headaches were a danger signs during pregnancy [25]. Only 7.9% women in India received information regarding danger signs during pregnancy and 8.7% in Tanzania [19,26].

In any ANC visit women should be informed of the signs of pregnancy complication. Only 19.6% women who completed primary school and 41.7% women who completed secondary school received information about signs of pregnancy in Indonesia [16]. Education is considered to be one reason influencing adequate ANC services among women in this area.

It is clearly a crucial problem, because the second cause of maternal death in Indonesia is eclampsia. This means it is critical to provide health education programs about the danger signs during pregnancy. Education is believed to be the most powerful influencing factor to increasing women's knowledge of a healthy pregnancy. Our study suggests that developing an education plan about danger signs is greatly needed.

### Where did women prefer to seek health care provider during pregnancy?

Our data indicated that women who preferred a TBA chose a TBA to check her pregnancy and they chose the government hospital as place of delivery. Titaley [27], reported that the role of the TBA was considered essential especially in remotes areas, and indicates that the women in developing countries especially in rural areas preferred TBA to check their pregnancy. Women were only seeking a professional health care provider if they had some kind of complication during their delivery.

Our findings showed that traditional beliefs were positively correlated with preferences of TBAs. Similarly, Koblinsky's [24] study found that preference for TBAs indicates tradition, because of TBA's interpersonal skill, special care, and respect for local customs. Likewise, the study by Titaley [27] in three districts of West Java, Indonesia using a purposive sampling method, focus group discussion and in-depth interviews as data collection found that in some communities, members perceived village midwives as too young and inexperienced, whereas TBAs were more mature, patient and caring compared with the midwife.

As expected, there was a strong association between family income and preference of TBAs and preference for midwives. This finding supports several other studies that confirmed family income is one of the factors influencing whether women decide to seek ANC [28]. In Indonesia pregnant women with higher family incomes had the highest percentage of adequate ANC utilization [29]. Cost was one of the main reasons women stated for using the services of TBA [27]. Poor women are most at risk of maternal mortality due to their lack of access to skilled care.

These findings indicate that women who held traditional beliefs need coaching in decision making about how to manage a healthy pregnancy. Health professionals should understand and respect the women's preferences then develop an educational plan during pre-pregnancy. This is important especially for primiparous and young women.

### Traditional beliefs and encouraging factors related to ANC visit

Traditionally, there has been discrimination towards women in decision-making, access to resources such as food, education and health care [30]. Many traditional communities across the country restrict a woman's food intake. Some women especially in rural areas agreed that following the traditional belief was necessary.

Women who were encouraged by their family to seek ANC had statistically significant higher traditional belief scores. This study supports Aikawa's [31] findings, where the majority of pregnancy women were encouraged and supported by their husband or their family members during pregnancy.

In Egypt, Indonesia, and Pakistan, beliefs include associating eclamptic convulsions with supernatural causes (e.g., spirit possession), requiring exorcism by faith healers rather than medical treatment [24].

Strong beliefs about the role of chance in determining the health of an infant had discouraged pregnant women from undergoing prenatal testing in Indonesia. Pregnant women admitted that they took their family advice without question because they trusted them and they wanted their family to be happy with them [32].

This study found no relationship between fewer ANC visits compared to traditional beliefs. It is possible that the five items about traditional beliefs were too few and additional items would increase the reliability and validity of questionnaire. There may also have been a selection bias because the subjects were recruited by village midwives and they tended to recruit women who came to their clinic. To explore information about the traditional belief context is more appropriate for further investigation through in-depth interviews. Health care providers should know the traditional beliefs of the women for whom they provide care. This will enable health care providers to have a more comprehensive understanding of the women's world view and they can provide appropriate decision- making and education. Encouragingly, better promotion of improving women's health will be an effective route to safe motherhood.

### Limitations and future plan

Although this study was limited by a small sample size, a researcher-developed questionnaire with minimal reliability and validity testing and selection bias, the findings still indicate some areas for attention. On the other hand, the ambiguous question whether headache is a normal sign during pregnancy needs to be a more clear statement if it is to be used for the next study. Based on our findings, psychometric development of the questionnaire and increasing the sample size are needed to generalize findings. Previous research has documented the need for increasing awareness of women's health especially increasing the number of ANC visits during pregnancy, intervention such as health education and health promotion. What this research contributes is a beginning understanding of determinants of Indonesian women's decision making so that health education can be more effective.

## Conclusions

In conclusion, the results of this study confirmed that parity was the main factor influencing women who received less than four ANC during pregnancy. An increased proportion attending ANC in pregnancy and an increased number of visits especially for primiparous women is important. Further, our study found that women who were encouraged by their family had higher traditional beliefs score. The results also indicated that traditional beliefs followed by family income dictated choice of care-giver. These findings were very important for understanding and exploring women's perceptions about the health services that they received.

## Competing interests

The authors declare that they have no competing interests.

## Authors' contributions

YA and SH were responsible for the study conception and design and the drafting of the manuscript. YA performed the data collection and YA and SH performed the data analysis. Both authors read and approved the final manuscript for submission.

## Pre-publication history

The pre-publication history for this paper can be accessed here:

http://www.biomedcentral.com/1471-2393/12/9/prepub
